# High-throughput replica-pinning approach to screen for yeast genes controlling low-frequency events

**DOI:** 10.1016/j.xpro.2021.101082

**Published:** 2022-01-13

**Authors:** Daniele Novarina, Fernando R. Rosas Bringas, Omar G. Rosas Bringas, Michael Chang

**Affiliations:** 1 European Research Institute for the Biology of Ageing, University of Groningen, University Medical Center Groningen, A. Deusinglaan 1, 9713 AV Groningen, the Netherlands

**Keywords:** Genetics, High Throughput Screening, Model Organisms, Molecular Biology

## Abstract

*Saccharomyces cerevisiae* is a leading model system for genome-wide screens, but low-frequency events (e.g., point mutations, recombination events) are difficult to detect with existing approaches. Here, we describe a high-throughput screening technique to detect low-frequency events using high-throughput replica pinning of high-density arrays of yeast colonies. This approach can be used to screen genes that control any process involving low-frequency events for which genetically selectable reporters are available, e.g., spontaneous mutations, recombination, and transcription errors.

For complete details on the use and execution of this protocol, please refer to (Novarina et al., 2020a, 2020b).

## Highlights

• Genome-wide screening to identify genes controlling low-frequency events in yeast
• Applicable to any such events for which there is a selectable genetic reporter
• Semi-quantitative estimation of low-frequency events rate for each strain

## Graphical abstract

**Figure:**
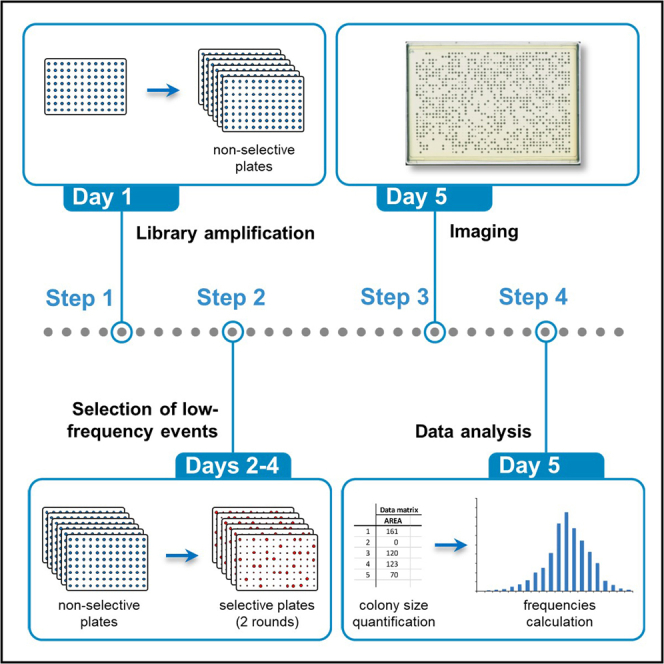

## Before you begin

**Figure 1:**
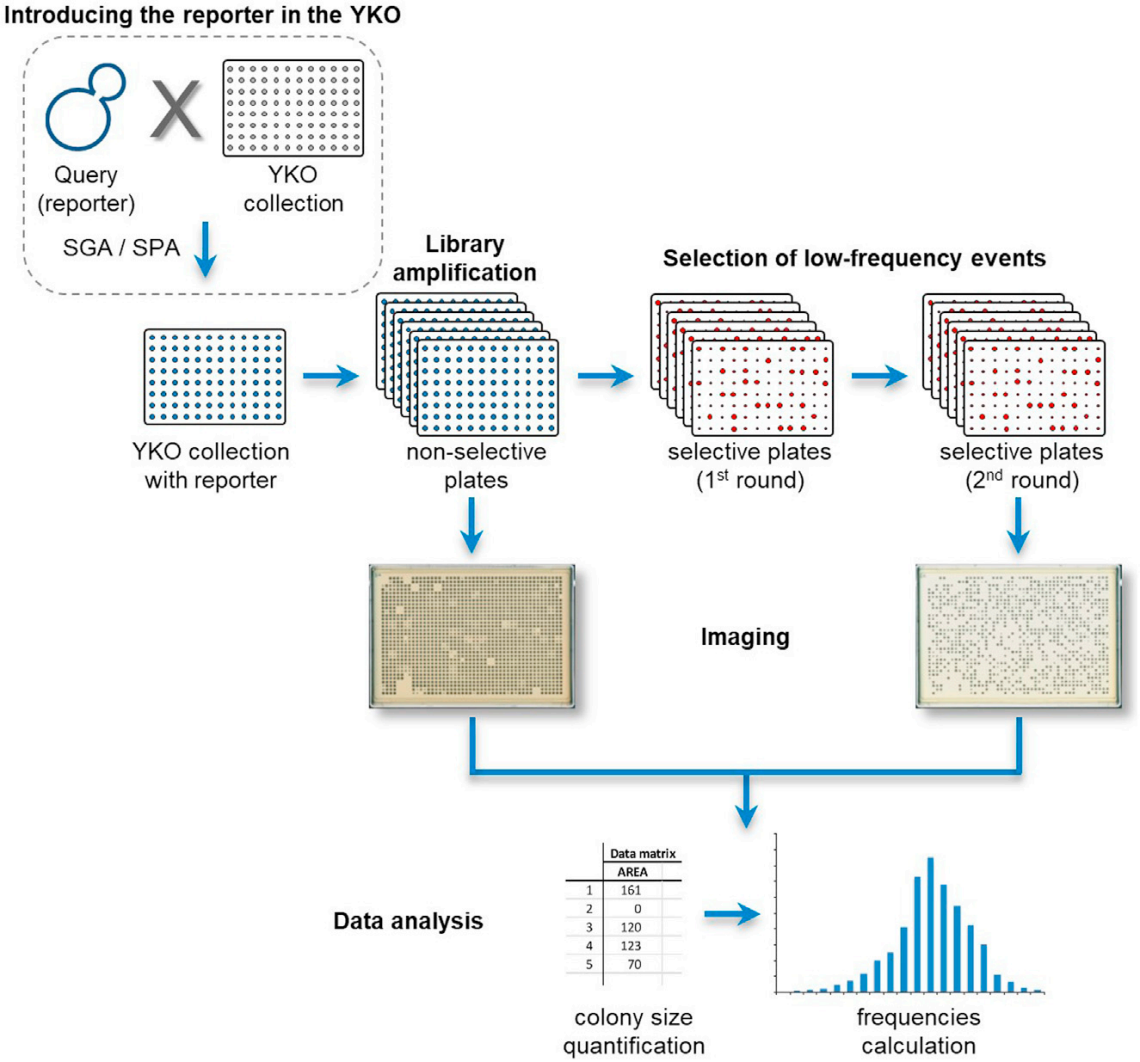
Schematic of the high-throughput replica pinning procedure.

This protocol describes a high-throughput replica pinning procedure to screen for genes affecting low-frequency events using an appropriate selectable genetic reporter (Figure 1). We have successfully applied this protocol to study the accumulation of spontaneous mutations during replicative aging using inactivation of the Mother Enrichment Program (Lindstrom and Gottschling, 2009) as a readout for spontaneous mutagenesis events (Novarina et al., 2020a), and to screen for genes that affect spontaneous direct-repeat recombination with the *leu2ΔEcoRI-URA3-leu2ΔBstEII* direct-repeat recombination reporter (Novarina et al., 2020b; Smith and Rothstein, 1999). As a standard example, we describe here the procedure for the spontaneous direct-repeat recombination screen (Novarina et al., 2020b). The protocol can be easily adapted to test other forms of genomic instability, or other low-frequency events, such as gross chromosomal rearrangements (Schmidt et al., 2006), inverted-repeat recombination (Rattray and Symington, 1994), transcription errors (Irvin et al., 2014; Strathern et al., 2012), transient loss of gene silencing (Dodson and Rine, 2015) and read-through at premature termination codons (Altamura et al., 2016), provided a genetically selectable reporter is available or could be developed. A suitable reporter has the following features: (i) it should be possible to select for the presence of the reporter (in the direct-repeat recombination example by selecting for the *URA3* marker); (ii) the reporter should allow the positive selection of the desired low-frequency event, based on the ability of cells to grow in selective conditions (in the direct-repeat recombination example, the functional *LEU2* gene resulting from spontaneous recombination allows growth on leucine-deprived plates); (iii) the reporter should be compatible with the SGA/SPA procedure (see introducing the reporter in the YKO collection); (iv) the reporter can be based on either auxotrophic or antibiotic-resistance markers (or a combination of both).

This protocol makes use of the yeast knockout (YKO) collection, an ordered library of yeast strains, where each of the ∼5000 non-essential *S. cerevisiae* open reading frames (ORFs) has been individually deleted by replacing it with the *kanMX* selectable marker, flanked by 20-bp molecular barcodes that allow individual mutant identification. The resulting strains can be arranged in a high-density array to facilitate high-throughput robotic manipulation (Giaever and Nislow, 2014). Before starting the high-throughput replica pinning procedure, it may be necessary to introduce the reporter of interest into the YKO collection.

***Note:*** The screen can be expanded by also including a library of hypomorphic alleles of essential genes (Kofoed et al., 2015; Li et al., 2011), or any other library of yeast strains, such as the DAmP (decreased abundance by mRNA perturbation) collection (Breslow et al., 2008) or the TetO_7_-promoter mutant collection (Hughes et al., 2000; Mnaimneh et al., 2004).

### Introducing the reporter in the YKO collection

**Timing: 1–4 weeks**

If the introduction of the reporter into the YKO library is necessary, this can be achieved in several ways. Two common methods are synthetic genetic array (SGA) (Tong and Boone, 2007) and selective ploidy ablation (SPA) (Reid et al., 2011).

1. Introduction of the reporter via SGA (for reporters that are chromosomally integrated).
  a. To construct the query strain for the SGA method, introduce the genetic reporter for the desired low-frequency event into the SGA starting strain Y7092 (Tong and Boone, 2007) using one of the following standard methods:
    i. PCR amplification and yeast transformation with the LiAc/SS carrier DNA/PEG method (Gietz and Schiestl, 2007).
    ii. Mating and tetrad dissection.
  b. Array the *MAT***a** YKO collection in 1536 format (on rectangular agar plates, up to 384 strains per plate with each strain in quadruplicate).
  c. Perform the SGA procedure (Kuzmin et al., 2016) to introduce the reporter from the query strain into the YKO collection.
2. Introduction of the reporter via SPA (for reporters on a plasmid or artificial chromosome).
  a. To construct the query strain for the SPA method, introduce the reporter-containing plasmid or artificial chromosome into the universal donor strain (UDS) (Reid et al., 2011) via transformation with the LiAc/SS carrier DNA/PEG method (Gietz and Schiestl, 2007).
  b. Array the *MAT***a** YKO collection in 1536 format (up to 384 strains per plate with each strain in quadruplicate).
  c. Perform the SPA procedure (Reid et al., 2011) to introduce the reporter-containing plasmid from the query strain into the YKO collection.

***Note:*** For selection of low-frequency events, choose a marker that is not already in use for the SGA or SPA procedure.

***Note:*** If you generate the SGA query strain by mating and tetrad dissection, make sure you start from a strain derived from the BY4741 genetic background (Brachmann et al., 1998), so that the query strain is isogenic to the YKO collection.

### Pilot experiment with positive and negative controls

**Timing: ∼1 week**

To properly calibrate the pinning conditions (see troubleshooting section), it is advisable to perform a pilot experiment with some reference test strains: at least a wild-type strain and a positive control (increased rate of the desired low-frequency event); if available, include also a control with a decreased rate of the desired low-frequency event.

3. Manually array the control strains on a plate in 384 format (i.e., 384 colonies per plate).
  a. Place a YPD plate in the “Source” position in the ROTOR (see key resources table and materials and equipment for details about pinning systems), leaving the target position empty.
  b. Perform a “fake pinning” step with a 384 format short pad.
    i. While touching the agar, the pins will leave a faint mark you can use as a guide to manually array your colonies.
    ii. Stop the pinning process after the pins detach from the “Source” plate.
  c. With the help of sterile pipette tips or toothpicks, manually array the control strains at the desired positions on the YPD plate.
4. Quadruplicate the 384 format array by automated robotically pinning the colonies in the 1536 format.
  a. Pin the colonies from 384 to 1536 format using the following ROTOR settings: Source plate: 384; Target plate: 1536; Pad: short pin 384; Program: Replicate Many; Revisit source: Off; Source offset: Off.
  b. Incubate the plates at 30°C for 1 day.

***Note:*** It is also possible to manually array the controls in 96 colony format and perform two subsequent automated pinning steps: the first to go from 96 to 384 format and the second (day after) from 384 to 1536 format.

5. Perform the whole screening procedure as described in the step-by-step method details section.

***Note:*** The only difference between the pilot experiment and the "real" experiment (see step-by-step method details) is that in the pilot a mini-array of strains that fit in one plate is analyzed (through the high-throughput replica-pinning procedure), while in the "real" experiment the procedure is performed with the whole YKO collection, arrayed on 14 plates.

6. Calculate the frequencies of colonies that underwent the desired low-frequency event for all the control strains as described in the step-by-step method details section.
7. If the frequency of the wild-type control is lower than 40% or higher than 60%, adjust the pinning procedure before performing the screen to obtain a pipeline that yields a wild-type frequency between 40% and 60% (see troubleshooting section).

***Note:*** To achieve a wild-type frequency between 40% and 60% is important only if you want to get hits with either an increased or a decreased rate of the desired low-frequency event. If you are interested in hits with only an increased or only a decreased rate, then the wild-type frequency does not need to be within the suggested interval.

### Preparation of plates

**Timing: 2–10 h**

8. For each plate of the reporter-containing YKO library, prepare at least “n” non-selective plates (NSP, in this case YPD), and “2n” selective plates (SP, in this case SD-leu), where “n” is the number of replica-pinning plates used for library amplification (step 1 of step-by-step method details). For instance, in the example described in this protocol the whole YKO collection is arrayed on 14 plates, and each plate is replica-pinned onto 6 non-selective plates, (“n” = 6). Therefore, the number of NSP required is 14 × 6 = 84, and the number of SP required is 14 × 6 × 2 = 168.
  a. Use standard recipes to prepare and sterilize agar medium (Treco and Lundblad, 2001).
  b. Pour 40 mL of medium in each PlusPlate on a level surface.
  c. Let the plates dry for 2 days at room temperature (20°C–25°C).

***Note:*** It is advisable to prepare more plates than strictly required (“n” NSP and “2n” SP) to have some backup plates in case of pinning errors or contamination.

***Note:*** Plates can be prepared in advance and stored at 4°C for up to (at least) 3 months, provided that they are placed in a plastic bag to prevent them from drying out. Before pinning, leave the plates on the bench for a few hours and make sure the surface is not wet due to condensation.

## Key resources table

**Table:** 

REAGENT or RESOURCE	SOURCE	IDENTIFIER
**Experimental models: Organisms/strains**		

*S. cerevisiae*: Yeast Knock-Out collection	EUROSCARF	N/A
*S. cerevisiae*: Y7092 (*MAT*α *his3Δ1 leu2Δ0 ura3Δ0 can1Δ::STE2pr-Sp_his5 lyp1Δ met15Δ0*)	Boone Lab (Tong and Boone, 2007)	N/A

**Software and algorithms**		

ImageJ	Schneider et al. (2012)	https://imagej.nih.gov/ij/download.html
ScreenMill Colony Measurement Engine	Dittmar et al. (2010)	https://sourceforge.net/projects/cm-engine/
R (*R-version >= 3.6.3*)	R Core team	https://cran.r-project.org
R packages: tidyverse (version 1.3.1) optparse (version 1.6.6) openxlsx (version 4.2.4)	R Core team	N/A
High-Throughput Replica Pinning script	This study	https://github.com/Chang-ERIBA/Protocol_HTRP

**Other**		

ROTOR-HDA pinning robot	Singer Instruments	N/A
RePads 384 Short	Singer Instruments	Cat#REP-004
RePads 1536 Short	Singer Instruments	Cat#REP-005
PlusPlates	Singer Instruments	Cat#PLU-003
64-bit computer running Linux, Mac OS or Window	N/A	N/A
Yeast Knock-Out collection collection keyfile in 384 format	N/A	N/A

## Materials and equipment

### Alternative equipment for colony pinning

We optimized this protocol using the ROTOR HDA pinning robot (for a demonstration about the robot please check this link: https://www.youtube.com/watch?v=SppI-ctJtOE), but it is possible to use other robotic colony pinning systems, such as the BioMatrix Colony Arrayer Robot (S&P Robotics, Inc.), as well as manual pin tools (V&P Scientific, Inc.).

### Equipment and software for data analysis

***Note:*** Instructions about how to install R and R studio, as well as the required R packages can be found in the Readme file of the High-Throughput Replica Pinning script.

## Step-by-step method details

### Library amplification on non-selective plates—Day 1

**Timing: 3 h**

Each plate of the reporter-containing YKO library is replica-pinned onto several non-selective plates in order to analyze multiple colonies per strain. For every position of the array, a colony will grow on the last plate of the pinning procedure if the low-frequency event occurred at that position, otherwise no colony will be present. Analysis of several independent colonies per strain allows us to calculate the fraction of colonies in which the desired low-frequency event occurred for a specific strain, giving a semi-quantitative estimate of the rate at which a specific low-frequency event occurs in each mutant strain of the YKO collection. Furthermore, this growth step on non-selective medium allows for spontaneous accumulation of the desired low-frequency event (in this case direct-repeat recombination) before the subsequent selection steps.

1. Replica-pin each plate of the library (in 1536 format) onto 6 YPD agar plates.
  a. Use the following ROTOR settings: Source plate: 1536; Target plate: 1536; Pad: short pin 1536; Revisit source: On; Source offset: Manual.

***Note:*** The number of replica plates at this amplification step is a choice of the user, and depends also on the density format in which the screen is performed. In the example described, 6 plates in 1536 format are a good compromise between analyzing several colonies for each strain (4 colonies × 6 plates = 24 colonies) and using a reasonably small number of plates. Increasing the number of plates could improve the accuracy of the screen, but would also increase the costs and the workload, while reducing the number of plates will reduce the workload and the costs at the expense of accuracy.

***Note:*** In order to have enough cells for pinning onto 6 target plates, it is preferable to touch each time a different region of the source colony with the pin. This can be achieved by manually adjusting the source pinning position using the Source > Offset > Manual option.

***Optional:*** One source plate is enough for pinning onto 6 target plates. If you need to use more than 6 target plates for library amplification, it is advisable to perform a pre-amplification step, where each library plate is pinned onto 2 target plates, each one serving as a source plate for (max) 6 target plates in the subsequent amplification step. Alternatively, the whole experiment can be performed in parallel with 2 independently constructed sets (named “set A” and “set B”) of the reporter-containing YKO collection.

2. Incubate the plates at 30°C for 1 day.

***Optional:*** In order to increase the number of analyzed colonies and/or reduce the number of plates, the whole screen could be performed in 6144 format (16 colonies per strain on each plate).

### Imaging non-selective plates—Day 2

**Timing: 1 h**

Images of the non-selective plates are taken to assess colony size before the selection starts.

**Figure 2:**
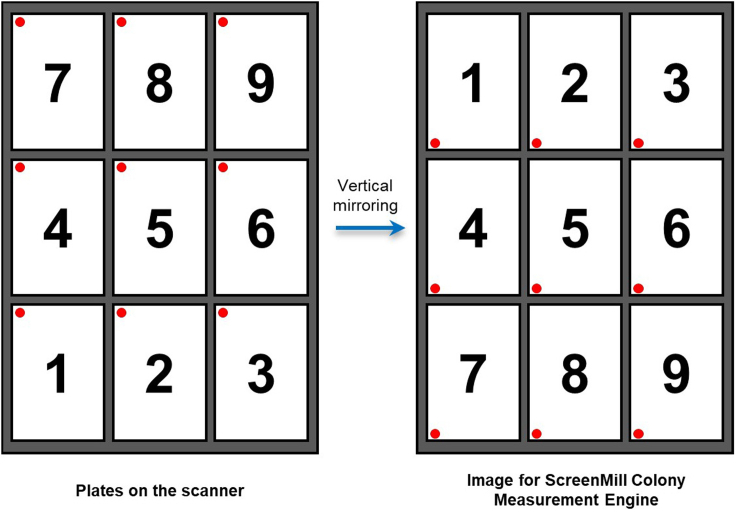
Plate orientation for imaging and analysis The plates should be arranged on the scanner in the order and orientation shown on the left (the red dot indicates the A1 position of each plate). Vertical mirroring then generates the image shown on the right, which correspond to the correct input format for ScreenMill Colony Measurement Engine.
4. Save images as TIFF files with a 300 dpi resolution.

***Note:*** While saving the images, use a name convention compatible with the ImageJ plugin ScreenMill Colony Measurement Engine (Dittmar et al., 2010), and the script that will be used for data analysis (see steps 11–20).

3. Image all YPD plates with a flatbed scanner using the plate order and orientation shown in Figure 2. We use a ScanMaker 9800XL scanner (MicroTek International, Inc.), scanning in 8-bit grayscale at 300-dpi resolution with the Transparent Media Adapter.

In this case: leuDRscreen1_NSP,1,2,3,4,5,6.tif

leuDRscreen2_NSP,1,2,3,4,5,6.tif

[…]

leuDRscreen14_NSP,1,2,3,4,5,6.tif

Where:leuDRscreen = screen ID (should NOT contain numbers)

1 = plate 1 of the YKO collection

NSP = non-selective plates (in this case YPD)

1,2,3,4,5,6 = the 6 parallel replicates of plate 1

### Pinning onto selective plates 1—Day 2

**Timing: 1.5 h**

Each plate is replicated once on the proper medium to select for the desired low-frequency events.

5. Replica-pin each YPD plate onto one SD-leu agar plate.
  a. Use the following ROTOR settings: Source plate: 1536; Target plate: 1536; Pad: short pin 1536; Revisit source Off; Source offset: Off.
6. Incubate the plates at 30°C for 2 days.

***Note:*** It is not necessary to take images of this set of selective plates, since the colonies will be very irregular in shape and difficult to analyze.

### Pinning onto selective plates 2—Day 4

**Timing: 1.5 h**

Second round of replica-pinning onto selective plates. This second round is needed to make the selection more stringent and thus obtain clearer results.

7. Replica-pin each SD-leu plate onto a new SD-leu agar plate.
  a. Use the following ROTOR settings: Source plate: 1536; Target plate: 1536; Pad: short pin 1536; Revisit source Off; Source offset: Off.
8. Incubate the plates at 30°C for 1 day.

### Imaging selective plates—Day 5

**Timing: 1 h**

Images of the selective plates are taken to assess colony size after the selection for the desired low-frequency events. The rationale here is that, at each position, a single low-frequency event that occurred during the non-selective growth (or before) will result in a fully grown colony, while if no event happened at that position the colony will not grow at all.

9. Image all plates from the second round of SD-leu selection with a flatbed scanner, using the same plate order and orientation as in step 3.
10. Save images as TIFF files with a 300 dpi resolution.

***Note:*** While saving the images, use the same name convention as before, where “NSP” is substituted with “SP” (= selective plates).

In this case: leuDRscreen1_SP,1,2,3,4,5,6.tif

leuDRscreen2_SP,1,2,3,4,5,6.tif

[…]

leuDRscreen14_SP,1,2,3,4,5,6.tif

### Image analysis

**Timing: 3 h**

Colony area is measured using the ImageJ software package (Schneider et al., 2012) and the ImageJ plugin ScreenMill Colony Measurement Engine (Dittmar et al., 2010). Colony area at each position is needed to assess the presence or the absence of a colony during the automated data analysis (see data analysis and frequencies calculation).

***Note:*** Contrary to what happens in fitness-based screens, differences in colony size (due, for instance, to the position of the colony on plate or the size of the neighboring colonies) are not a problem in this protocol, because the final output that is scored is essentially binary (colony or no colony).

***Note:*** The ImageJ plugin ScreenMill Colony Measurement Engine can be found here https://sourceforge.net/projects/cm-engine/. The plugin file (cm engine_ImageJ1.63.txt) should be saved in the “macros” folder of ImageJ.

11. Install the ImageJ plugin ScreenMill Colony Measurement Engine as a macro.
  a. Plugins>Macro>Install.
  b. Select the file “cm engine_ImageJ1.63.txt” from the macros folder.
12. Open the Colony Measurement Engine.
  a. Plugins>Macro>ScreenMill - CM Engine [c].
13. Choose Parent Directory: select the folder containing the images of the non-selective plates.
14. Use the following setting from the “Screen Information” menu.
  a. Fine-crop mode: Automatic.
  b. Colony measurement method: Standard.
  c. Plate density: 1536.
  d. File name to save measurements to: chose the file name you prefer (for instance “leuDRscreen_NSP_colonyAreas”).
  e. Running mode: Standard.
15. Repeat the same procedure to quantify the images of the selective plates.

***Note:*** During the image analysis, you are occasionally asked to verify (and adjust) the plate fine cropping, and to verify and manually remove possible artifacts on the plate (such as scratches or contamination) that interfere with colony quantification. Please follow the instructions from the pop-up windows to adjust the cropping and remove artifacts. For more information, please check the instructions in the electronic supplementary material from Dittmar et al. (2010).

***Optional:*** It is also possible to analyze the images from non-selective plates and selective plates at the same time by placing NSP and SP image files in the same folder (steps 11–14).

### Data analysis and frequencies calculation

**Timing: 15 min**

This section describes an automated script to analyze the raw colony size dataset. The working script calculates the frequency of SP/NSP colonies for each strain, and generates an Excel file containing a ranked list of genes according to this frequency.

16. Download the High-Throughput Replica Pinning script available on GitHub: https://github.com/Chang-ERIBA/Protocol_HTRP

***Note:*** This R script will require the following packages alongside with R-version 3.6.3 or later (for more information please check the Readme file in GitHub Repository):

tidyverse (version 1.3.1)

optparse (version 1.6.6)

openxlsx (version 4.2.4)

17. Open a “**Terminal**” in the working directory where the scripts *High_Replica_Pinning_Tools.R* and *High_Replica_Pinning.R* are present*.*
18. To execute the program, paste the following command:

Rscript High_Replica_Pinning.R -i <Inputfile> -k <Keyfile> -O <Output_Dir> --Filter <filterValue> --Median_NSP <MedianRatioNSP> --Median_SP <MedianRatioSP>

19. Fill in the required input files and parameters.
  a. Required input files:
    <Inputfile>: File with the colony areas data. Generated in the section image analysis (steps 11–15).
    ***Note:*** If the image analysis (steps 11–15) was performed separately for NSP and SP plates, you need to combine the two output .txt files into one single file by simply copying and pasting the content of one file to the bottom of the other.
    <Keyfile>: library Keyfile in .csv and 384 format.
    ***Note:*** The library Keyfile must contain the following columns:
    *Plate* #: Numbers
    *Row*: Letters from A to P
    *Column*: Numbers from 1 to 24
    *ORF*: Systematic names of *S. cerevisiae* genes
    *Gene*: Gene names
    Mutation (optional; if present, it will be used to group and calculate “*Total colonies*” at the “*Grouped*” tab in the final document: see step 20).
    ***Note:*** If the column *Mutation* is absent, the grouping will be by *ORF* and *Gene*. If the column *Mutation* is present (such as in the temperature-sensitive collection, where different mutations can be present for the same gene, and the same mutant might be repeated at different positions of the collection), the grouping will be by *ORF*, *Gene* and *Mutation*.
  b. Optional parameters:

**Table:** 

<Output_Dir>: Output directory. If not added, a new folder named “Output” will be created.
<filterValue>: Numeric value used as a threshold to filter and exclude strains with a total number of colonies on non-selective plates less than <filterValue>. By default, the threshold is **10**.
<MedianRatioNSP>: Numeric value between 0 and 1 that indicates the percentage of the NSP colony size median used as a size threshold to calculate the number of colonies on non-selective plates. By default, the value is **0.5** (i.e., only colonies with a pixel area greater than 50% of the median NSP pixel area will be scored).

    ***Note:*** The number of colonies that pass the threshold is reported in column “*colonies_NSP*” in the final Excel file.
    <MedianRatioSP>: Numeric value between 0 and 1 that indicates the percentage of the NSP colony size median used as a size threshold to calculate the number of colonies on selective plates. By default, the value is **0.2** (i.e., only colonies with a pixel area greater than 20% of the median NSP pixel area will be scored).
    ***Note:*** The number of colonies that pass the threshold is reported in column “*colonies_SP*” in the final Excel file.
    ***Note:*** The default <MedianRatioSP> is smaller than the default <MedianRationNSP> because colonies on SP plates are often smaller than on NSP plates.

20. Run the script. This will generate one Excel file named:

<ColonyFileName>_<MedianRatioNSP>_<MedianRatioSP>_<filterValue>_<Date>.xlsx

The file contains the following tabs:

**Table:** 

*Raw*: Represents the raw data ordered by 1536 position. The percentage of SP/NSP colonies (frequency) is shown in column “*Freq_percent*”.
*Raw*-*Ordered*: The raw data is ordered by ORF name.
*Ordered*: The pooled data for each quadruplicate strain is ordered by gene/ORF name.
*Ranked*: The data is ordered from the highest to lowest frequency.
*Filtered*: Positions with less than <filterValue> and empty positions in the <Keyfile> are excluded.
*Grouped*: Genes with the same name at ORF and gene columns are grouped and counted together after being filtered (in case the same strain is present at several positions of the 384-format collection, they will be grouped together).

***Note:*** If the collection keyfile contains a “*Mutation*” column (as in the temperature-sensitive collection), the list will be grouped by ORF, gene and mutation names.

***Note:*** The Readme file of the script contains two examples of how to use the script with a test dataset available in the script repository website.

## Expected outcomes

**Figure 3:**
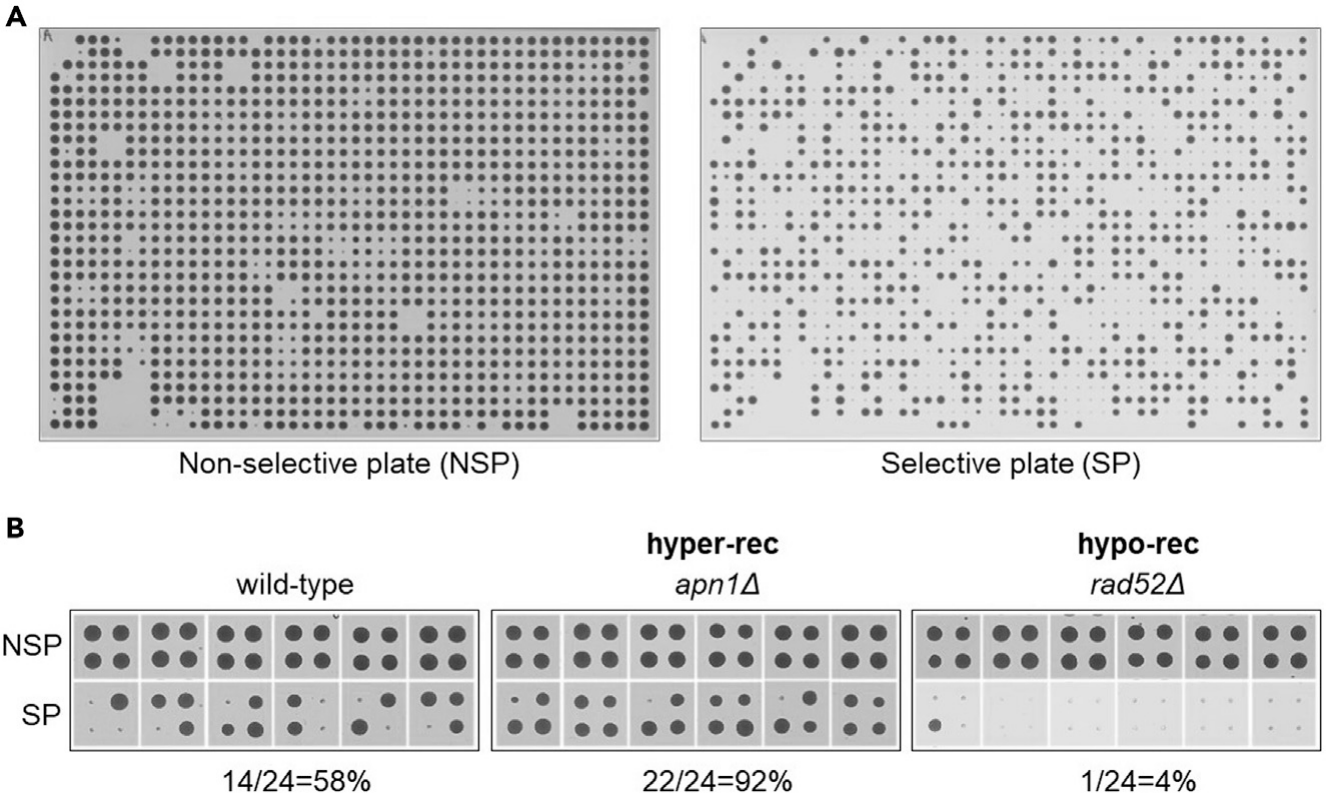
Examples of expected outcomes (A) Example of a non-selective plate (NSP) and the corresponding selective plate (SP). (B) All 24 replica-pinned colonies of a wild-type control strain and of two selected mutants with increased (*apn1Δ*) and decreased (*rad52Δ*) recombinant frequencies from a screen for genes affecting spontaneous direct-repeat recombination (Novarina et al., 2020b) are shown. Colony frequencies for each strain are reported below the corresponding images.

An example of a non-selective plate (NSP) and the corresponding selective plate (SP) from the spontaneous direct-repeat recombination screen (Novarina et al., 2020b) is shown in Figure 3A. From the same dataset, all 24 replica-pinned colonies of a wild-type control strain and of two mutants with increased and decreased recombinant frequencies are shown in Figure 3B. To allow proper detection of genes causing both an increase and a decrease of the desired low-frequency event, the colony frequency of the wild-type control strain should be roughly between 40% and 60%. This frequency can be lower if the screen is aimed at identifying only mutants with an increased frequency, and higher if the screen is aimed at identifying only mutants with a decreased frequency of a specific low-frequency event. A pilot experiment with a few control strains is recommended before performing the whole screen (see before you begin). To adjust the pinning conditions to obtain the desired wild-type colony frequency, please check the troubleshooting section.

The final outcome of the high-throughput replica pinning procedure described in this protocol is a list of all tested genes, ranked according to colony frequency for the desired low-frequency event. A p value cutoff for the genes at the top and the bottom of the ranked list can be set by Chi squared test or Fisher’s exact test based on the observed frequencies on NSP and SP plates in the whole screen. Alternatively, bioinformatics tools can be used, such as the Cutoff Linked to Interaction Knowledge (CLIK) algorithm (Dittmar et al., 2013).

***Note:*** If the screen is performed with a barcoded library, the identity of individual strains at specific positions can be verified by barcode sequencing (Mcmahon et al., 2011).

## Limitations

The high-throughput replica pinning screening methodology has been developed and validated for low-frequency events that occur at a rate in the order of 10^−7^ to 10^−5^ (events per cell division) in a wild-type strain. Even though the procedure can be adapted to higher or lower rates (see troubleshooting section), this protocol is not suitable to study low-frequency events that occur at a rate several orders of magnitude higher or lower.

It has to be taken into account that the frequencies calculated for the pinned colonies are a semi-quantitative indication of the rate at which the desired low-frequency event occurs, but there is no linear relation between the rate of occurrence of the low-frequency event and the colony frequencies measured in this protocol.

As with most screens performed with the YKO collection, the methodology described in this protocol has some intrinsic technical limitations. In particular, mating-deficient mutants will be lost during the SGA or SPA procedure to insert the reporter into the YKO collection, and cannot therefore be analyzed. Similarly, slow-growing strains might be also lost during the pinning procedure. Moreover, several strains of the YKO collection were created in an *HIS*^*+*^ genetic background (Winzeler et al., 1999). For this reason, they are not suitable for the SGA procedure, and have to be removed from the final dataset. Furthermore, a set of strains from the YKO collection were found to carry an additional mutation in the mismatch repair gene *MSH3* (Lehner et al., 2007). These strains display an elevated spontaneous mutation and recombination rate, independently of the identity of the intended gene deletion. These strains should be discarded from the final dataset of screens for genome instability-related low-frequency events. On the other hand, these strains can be used as a set of positive controls, for instance, in case of screens for genes affecting spontaneous mutagenesis or recombination (Novarina et al., 2020a, 2020b).

Finally, as in the case of all high-throughput screens, the data obtained by screening with the high-throughput replica pinning procedure will contain false positives and false negatives. While the dataset is useful to give a general overview of the genes and pathways controlling a specific low-frequency event, single candidate genes need to be individually validated by fluctuation tests (Foster, 2006; Hall et al., 2009; Luria and Delbrück, 1943).

## Troubleshooting

### Problem 1

The frequency of wild-type colonies on selective medium is too high (steps 7–10 of step-by-step method details). If the wild-type frequency is higher than ∼70%, the distribution of the frequency for the whole collection will be skewed towards high values, making it difficult to identify strains with an increased frequency compared to the wild-type.

### Potential solutions

Reduce the number of cell divisions on non-selective plates. This can be achieved in several ways:

Reduce the time of incubation of non-selective plates (can be as short as 6 h) prior to replica-pinning on selective media.

Skip the non-selective step and perform the library amplification step during the last selection step of the SGA or SPA procedure (see introducing the reporter in the YKO collection). These plates should be imaged before replica-pinning on selective plates for the low-frequency events and used as the comparison control (“NSP” plates in the High-throughput Replica pinning script).

An alternative (and complementary) strategy is to reduce the number of cells per colony, for example by performing the library amplification step with a pad format that deposits fewer cells on the agar surface (for instance pinning colonies in 384 format using a 1536 pad, or pinning colonies in 1536 format using a 6144 pad; in this case, it is important to make sure that only one out of four pins touches the source colony). If the amplification step is performed on non-selective plates, you should also reduce the time of incubation of non-selective plates prior to replica-pinning on selective media.

***Note:*** The number of divisions required to achieve a 40%–60% WT frequency depends on the kind of low-frequency event investigated and the specific reporter chosen, and thus has to be empirically determined (see pilot experiment with positive and negative controls).

**Figure 4:**
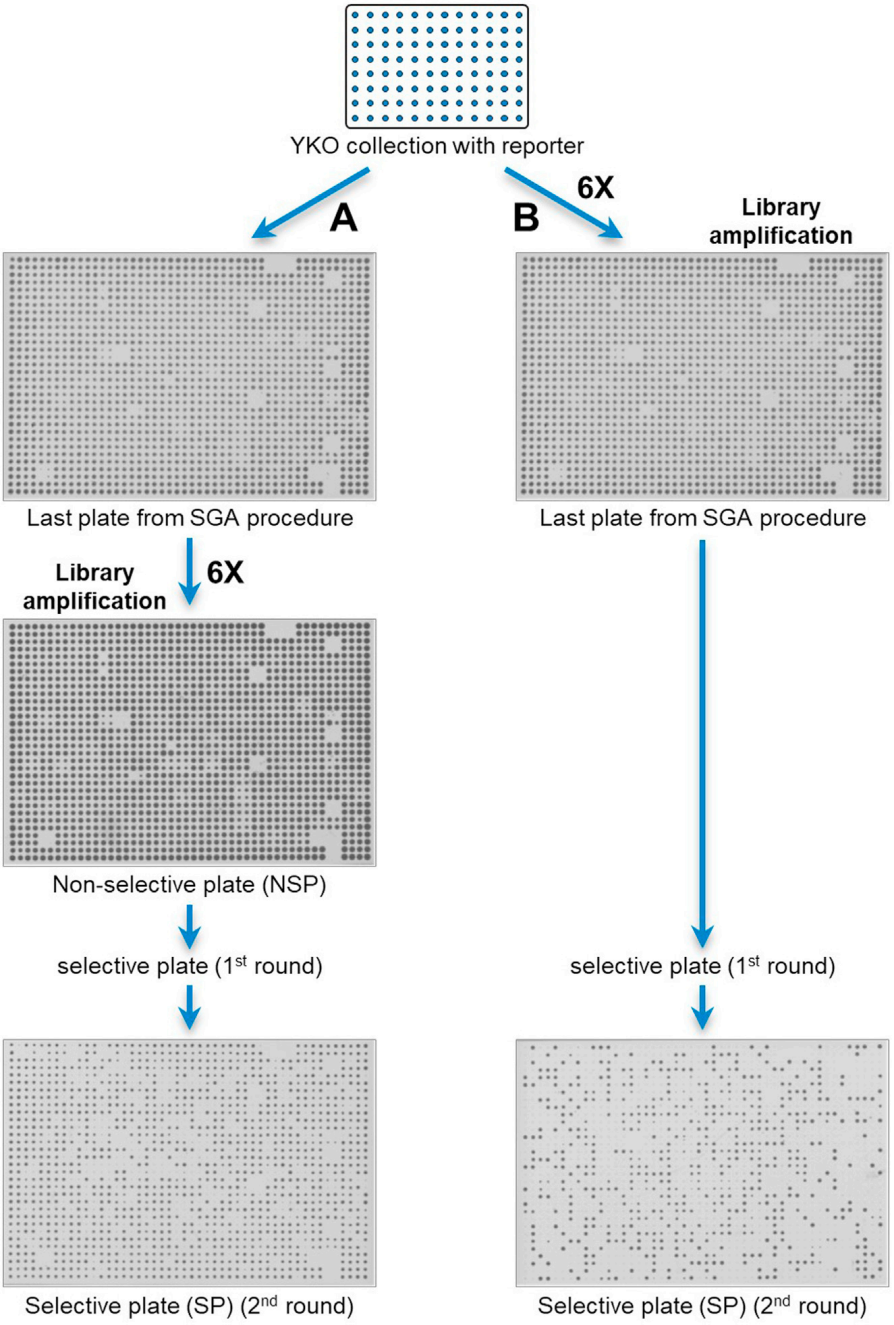
Example of troubleshooting to adjust the colony frequencies (A) According to the standard procedure, the library amplification step is performed on non-selective plates (A), followed by pinning onto selective plates to detect the desired low-frequency events. (B) To reduce the colony frequencies, the library amplification step can be performed on the last SGA selection plates, followed by pinning directly onto selective plates (B). See text for further details.

***Note:*** The approaches described in this section can be combined with the standard procedure in case the colony frequencies for strains with increase and decreased low-frequency events compared to the wild type is very big. For instance, the library (set A) can be amplified on non-selective plates according to the standard protocol to detect strains with a decreased rate of the desired low-frequency events, and in parallel, the same library (set B) can be amplified on the last SGA selection plates and then replica-pinned directly onto selective plates to identify strains with an increased rate of the same low-frequency events (Figure 4).

### Problem 2

The frequency of wild-type colonies on selective medium is too low (steps 7–10 of step-by-step method details). In this case, virtually no low-frequency events will be detected for most of the strains of the YKO collection.

### Potential solutions

Increase the number of plates from the library amplification step. Even if the frequency will remain low, the absolute number of colonies that can grow on selective plates will be higher, increasing the chance of detecting more subtle differences between the wild-type and the tested strains.

Perform several subsequent rounds of replica pinning on non-selective plates before selecting for the desired low-frequency event.

Perform the screen in a “sensitized setting”, such as in the presence of a chemical or in a genetic background that increases the basal level of the desired low-frequency event.

### Problem 3

Slow-growing strains are lost during the SGA/SPA procedure or they do not pass the threshold for the minimal number viable colonies on NSP plates, specified by the <filterValue> parameter of the High-Throughput Replica Pinning script (steps 1–4 of step-by-step method details).

### Potential solution

Array all slow-growing strains on a 384-format plate (some versions of the YKO collection already include one extra plate containing all slow-growing strains), and use this plate to perform the whole protocol, adding one extra incubation day at each plate incubation step.

### Problem 4

The amount of potential hits of the screen that pass the chosen cutoff (see expected outcomes section) is too big for direct validation via fluctuation test, or upon direct validation of the first hits, a lot of false positives are detected.

### Potential solutions

Change the criterion for the choice of the cutoff to reduce the number of hits to be validated

Create a mini-array by placing all the candidate hit strains from the YKO onto 1–2 plates, perform the SGA/SPA procedure again to introduce the reporter into the candidate strains, and then perform a first round of (rough) validation through the patch-and-replica-plating method (Novarina et al., 2020b). Subsequently, perform a second round of validation via fluctuation tests for the strains that pass the first round of validation.

### Problem 5

The High-Throughput Replica Pinning script gives an error message. This can be due to several reasons, which are discussed below.

### Potential solutions

If the error message says “Some plates do not have a correct name: SP or NSP.”, there is an error in the plate names in the <Inputfile> with the colony areas data. This is probably due to improper naming of the plate images (steps 4 and 10). Please check the names of the plate images and format them correctly. Then repeat the image quantification and data analysis (steps 11–20). Alternatively, you can correct the mistake directly in the <Inputfile>, and then run the script again (steps 17–20).

If the error message says “There is an error in the keyfile.”, the format of the <Keyfile> is not correct. This could mean that one of the required columns is missing or that the content of some columns is not formatted correctly. Please verify the <Keyfile> format and correct it according to the script requirements (see Notes to step 19). It is important to check that the keyfile is separated with commas (CSV format).

The script gives just a general “Error” message. This could happen if a required input file is missing from the script execution command, or if some optional parameters are not formatted correctly (for instance a <MedianRatioNSP> or <MedianRatioSP> is >1). Please verify that the input files and parameters are correctly inserted in the command to execute the script (step 19).

An “Error” can also occur if the script was already used to generate another <Outputfile> on the same day. In this case a file with the same name as the <Outputfile> is already present in the “Output” folder. This can be easily solved by renaming the already existing output file, or moving it to another folder. Alternatively, it possible to choose another <Output_Dir> while running the script (steps 19–20).

## Resource availability

### Lead contact

Further information and requests for resources and reagents should be directed to and will be fulfilled by the lead contact, Michael Chang (m.chang@umcg.nl).

### Materials availability

This study did not generate new unique reagents.

## Data Availability

This study did not generate datasets. The code generated during this study is available at Protocol_HTRP repository (https://github.com/Chang-ERIBA/Protocol_HTRP).
